# Hyperbilirubinemia and Neurodevelopmental Outcome of Very Low Birthweight Infants: Results from the LIFT Cohort

**DOI:** 10.1371/journal.pone.0030900

**Published:** 2012-01-27

**Authors:** Gaël Mazeiras, Jean-Christophe Rozé, Pierre-Yves Ancel, Gaëlle Caillaux, Anne Frondas-Chauty, Sophie Denizot, Cyril Flamant

**Affiliations:** 1 Department of Neonatal Medicine, St Leon Hospital, Bayonne, France; 2 Department of Neonatal Medicine, Nantes University Hospital, Nantes, France; 3 National Institute of Health and Medical Research CIC004, Nantes University Hospital, Nantes, France; 4 “Loire Infant Follow-up Team” (LIFT) Network, Pays de Loire, France; 5 National Institute of Health and Medical Research Mixed Research Unit S149, Federal Institute of Research 69, Epidemiological Research Unit on Perinatal and Women's Health, Tenon University Hospital and Pierre and Marie Curie University, Paris, France; University of Utah School of Medicine, United States of America

## Abstract

**Background:**

Bilirubin-related neurotoxicity is an important clinical issue in very low birthweight (VLBW) infants, and the existing literature is inconsistent.

**Objective:**

To analyze the relationship between maximal serum unconjugated bilirubin levels (SBL) and neurodevelopmental outcome at 2-year corrected age in VLBW infants.

**Methods:**

Phototherapy was initiated in all infants born before 33 weeks of gestation, according to Maisels' recommendations. Neurodevelopmental assessment at 2-year corrected age was performed in all infants that survived. SBLs collected during the first week of life were used to define three tertiles of max-SBL. The first tertile corresponded to infants with the lowest max-SBL.

**Results and Conclusions:**

A total of 724 infants were included in the study, and among them, 631 (87%) were evaluated at two years old. The infants of the first tertile were younger and smaller than the infants of the other two tertiles, in accordance with Maisels' recommendations for very small infants. No difference in the risk of impaired functional outcome among the three groups was observed. However, among infants weighing less than 1001 g, those in the third tertile had a poorer neurodevelopmental prognosis as compared to those in the second tertile (adjusted odds ratio = 6.8, 95% CI: 1.2–36.7, p = 0.03). Considering the results obtained, we propose 196 µmol/L (11.5 mg/dL) when birthweight varies between 1001 and 1500 g, and 170 µmol/L (9.9 mg/dL) when birthweight is less than 1001 g, as recommended max-SBLs (defined as maximal levels of 95^th^ percentile curves of SBLs in infants with an optimal outcome). When Maisels' recommendations were applied, max SBLs were higher in 8% of infants weighing 1001–1500 g and in 15% of infants weighing less than 1001 g. Our data seems to validate Maisels' recommendations in the overall population of infants born before 33 weeks of gestation, but not in infants weighing less than 1001 g.

## Introduction

Despite showing a potentially protective antioxidant effect, unbound bilirubin is neurotoxic, and accumulates in the basal ganglia and in numerous brainstem nuclei [1]–[3]. Indeed, a high level of unbound bilirubin can lead to acute encephalopathy and kernicterus, although good management can reduce the occurrence of these complications [1]. The management of bilirubin in infants born before 35 weeks of gestation has been well-documented [1]. However, little is known about hyperbilirubinemia in very low birthweight (VLBW) infants.

Premature infants are at a special risk of elevated bilirubin production, lower hepatic conjugation, lower albumin binding capacity and increased central nervous system sensitivity to unbound bilirubin, depending on birthweight [3]–[4]. These factors increase the risk of neurosensory injury, and cases of acute encephalopathy have been reported in preterm infants with total bilirubin levels lower than those usually reported at term [4]–[5]. Given these considerations, specific thresholds of bilirubin levels for initiating phototherapy in premature infants have been suggested [4], [6]–[7].

Nevertheless, few studies have evaluated the neurodevelopmental benefits of these recommendations. A first work reported a correlation between maximal total bilirubin level and disability at 2 years of age among VLBW infants, despite hyperbilirubinemia treatment according to Maisels' recommendations [7]–[8]. In another study, O'Shea *et al.* recommended a threshold level of 86 µmol/L (5 mg/dL) of total bilirubin for starting phototherapy in infants weighing less than 1500 g. No association was found between maximal total bilirubin and neurodevelopmental outcome at 12-month corrected age [9]. Two other studies reported an association between maximal total bilirubin level and impaired neurodevelopmental outcome in VLBW infants at 18- and 22-month corrected ages [10]–[11], although the phototherapy procedure was not specified. Oh *et al.* showed that an increasing level of unbound bilirubin in unstable neonates was associated with death and adverse neurodevelopmental outcome at 18 and 22 months of corrected age. Paradoxically, the authors found an association between decreasing levels of total bilirubin and poor outcome in stable infants [12]. Recently, Moll *et al.* reported the cases of two extremely low birthweight (ELBW) infants with kernicterus, and questioned whether current phototherapy guidelines are appropriate for high-risk ELBW infants [13].

Given the lack of validated recommendations regarding phototherapy in VLBW infants, the aim of this study was to assess the potential relationship between bilirubin levels during the first days of life and neurological outcome in a cohort of preterm infants reaching 2 years of age.

## Methods

### Study population and data collection

All surviving infants born before 33 weeks of gestation in Nantes University Hospital (France) between January 2003 and December 2006 were enrolled in the LIFT cohort and included in this study. The cohort was registered at the French CNIL (Commission Nationale de l'Informatique et des Libertés n°851117) ethics committee in order to collect clinical data from patients' records. Specific approval to use the data in this study was obtained from the Institutional Review Board of the University Hospital of Nantes. The children's parents provided written informed consent, which was obtained in each case before inclusion in the LIFT cohort and before using the data. All potential participants who declined to participate or otherwise did not participate were not disadvantaged in any way by not participating in the study. The day of the birth was considered Day 1. Initial data were prospectively collected. During hospitalization, phototherapy was started following Maisels' recommendations [7], which were described in a notebook for all clinicians. According to these recommendations, phototherapy was started in infants with a birthweight lower than 1500 g and with low serum unconjugated bilirubin levels (SBLs). Biochemical data were collected from the prospectively entered hospital biochemistry database. The child's file number allowed for linkage between biochemical and clinical data. SBLs were collected for the first week of life, and the maximal serum unconjugated bilirubin level (max-SBL) was recorded for each child.

### Patient assessment

After parental consent was obtained, the infants were enrolled in our Loire Infant regional follow-up network [14] and evaluated at 2-year corrected age. Neurodevelopmental assessment included physical examination by a trained pediatrician and psychomotor evaluation by a psychologist. Neuromotor function was regarded as non-optimal in cases of cerebral palsy or when milder signs consistent with impaired independent walking were present at 2-year corrected age. Psychomotor development was assessed with the revised Brunet-Lézine test [15]. The test was performed by a specialized psychologist and evaluated four developmental areas: fine motor skills, social skills, language skills, and posture and gross motor adaptation [16], allowing the calculation of four separate scores plus an overall developmental score. Values lower than 85 indicated a non-optimal psychomotor development. The infants who were not able to take the Brunet-Lézine test because their neurologic impairment was too severe were included in the “non-optimal psychomotor development” group. When a psychological evaluation could not be conducted, outcome was assessed with the Ages and Stages Questionnaire (ASQ) [17]. This parent-completed developmental questionnaire is divided into five areas that assess a child's development: communication, gross motor skills, fine motor skills, problem solving and personal/social skills. The ASQ has been recently validated by comparison with the revised Brunet-Lézine test and with other formal psychometric assessment tools [18]–[20]. An abnormal ASQ was considered when patients did not meet at least two of the five areas and defined non-optimal psychomotor development. Children with non-optimal neuromotor and/or psychomotor assessments were considered as having impaired functional outcome.

### Statistical analysis

Three tertiles of max-SBL were defined using SPSS 15.0 (Chicago, I). The infants from the first tertile constituted the reference group. Functional outcome at 2 years of age was analyzed as a binary variable. The analysis was conducted in four steps. In the first step, the relationship between max-SBL tertiles and clinical characteristics was analyzed. In the second step, the relationship between the latter variables and 2-year neurodevelopmental outcome was assessed. The χ^2^ test, with Yates' correction if necessary, and analysis of variance were used to compare the characteristics of infants and 2-year outcome among the three tertiles. In the third step, the association between max-SBL and neurodevelopmental outcome was evaluated (crude association). A logistic regression was performed to adjust for confounding variables. Adjusted odds ratios (aOR) for neuromotor and psychomotor impairment according to max-SBL were calculated. In the fourth step, the relationship between max-SBL and 2-year neurodevelopmental outcome was assessed in three subpopulations: infants with birthweight under 1001 g, between 1001 and 1500 g, and over 1500 g. For all subpopulations, three progressive curves of SBLs were created, which represented the median distribution of SBLs in the first week of life for each tertile. We defined a recommended max-SBL for each subpopulation, which consisted of the maximal level of the 95^th^ percentile curve of SBLs in children with a normal neurodevelopmental outcome at 2 years. The results were expressed as crude and aOR, including 95% confidence intervals (CIs), for adverse neurodevelopmental outcome associated with increased max-SBL. Moreover, to increase the sensibility of our analyses, we analyzed the correlation between ASQ and max-SBL, adjusted for gestational age (GA), in the overall population and in each subgroup according to birthweight. All tests were two-tailed, and the significance level was set at 0.05.

## Results

### Study population and follow-up

**Figure 1:**
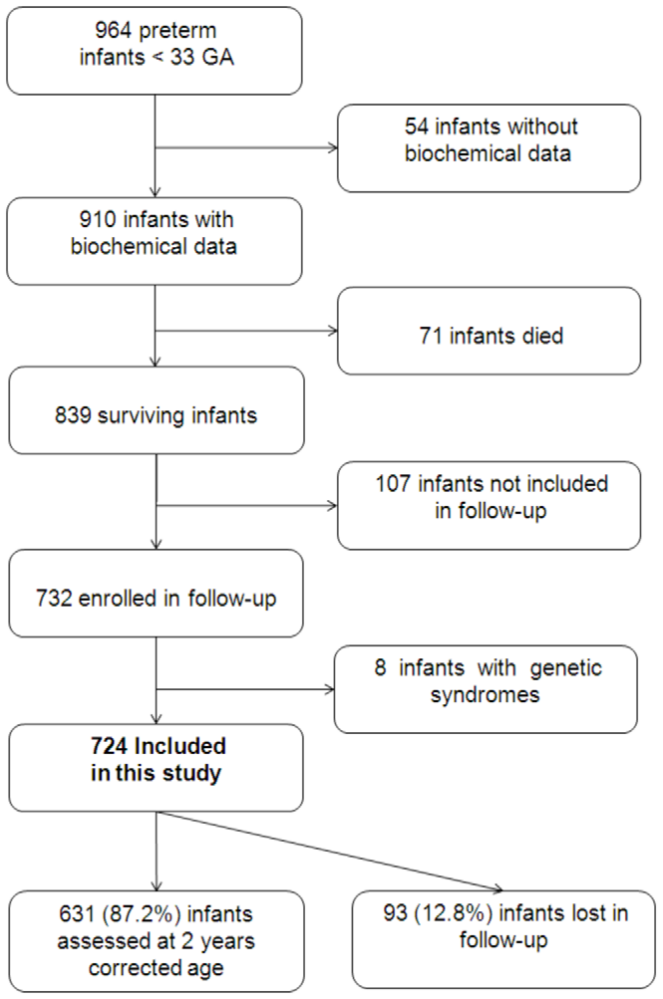
Characteristics of the study population.

Of the 964 preterm infants born before 33 weeks of gestation during the four years of the study period, perinatal data was linked with biological data for only 910 of the infants. Of these 910 infants, 71 died during hospitalization and 839 survived. Max-SBL was significantly lower in the infants who did not survive (108±62 *vs.* 152±59 µmol/L, p = 0.001, respectively). Among the 839 surviving infants, 732 were enrolled in the regional network. Eight infants with genetic syndromes were excluded from the analysis. A total of 724 infants were finally included in the analysis (Fig. 1). There was no significant difference between infants who were included (n = 724) and who were not included (n = 115) regarding GA (29.7±2.0 *vs.* 29.9±2.0 weeks, p = 0.27) and birthweight (1340±460 *vs.* 1400±370 g, p = 0.16). Max-SBL was significantly lower in infants who were not included in the analysis (130±87 µmol/L vs. 156±51 µmol/L; p = 0.001, respectively).

### Risk factors for elevated max-SBL

The total number of serum unconjugated bilirubin measurements was 2,746 (Table 1). The infants in the two last tertiles had significantly more blood analyses and a significantly greater max-SBL than infants in the first tertile. Infants in the first tertile had a significantly lower GA and lower birthweight than infants in the other two tertiles (Table 1). Furthermore, nosocomial sepsis, bronchopulmonary dysplasia, hemodynamic failure and patent ductus arteriosus were significantly more frequent among the younger and smaller infants of the first tertile. There was no difference in the frequency of radiological assessments or radiological findings between the infants in the different tertiles, nor in the follow-up or outcome information.

**Table 1:** Bilirubin determination and characteristics of preterm infants according to different tertiles of maximal serum non-conjugated bilirubin level.

Characteristics	First Tertile (N = 227)	Second Tertile (N = 250)	Third Tertile (N = 247)	p-value
**Bilirubin determination**				
Number of serum non-conjugated bilirubin determinations per infant during the first week of life, mean (SD)	2.7 (1.4)	4.5 (1.3)	4.6 (1.1)	0.001
Maximal serum non-conjugated bilirubin level, µmol/L, mean (SD)	100.6 (35.7)	156.1 (9.5)	208.1 (33.5)	0.001
Age of maximal serum non-conjugated bilirubin determination in days, mean (SD)	3.8 (1.2)	4.4 (1.2)	4.5 (1.0)	0.001
**Infants' characteristics**				
Gestational age (GA)				
24–26 weeks (%)	36 (15.9)	15 (6.0)	7 (2.8)	0.001
27–28 weeks (%)	60 (26.4)	51 (20.4)	25 (10.1)	
29–30 weeks (%)	57 (25.1)	104 (41.6)	61 (24.7)	
31–32 weeks (%)	74 (32.6)	80 (32.0)	154 (62.3)	
Birthweight				
<1001 g (%)	95 (41.9)	47 (18.8)	25 (10.1)	0.001
1001–1500 g (%)	77 (33.9)	147 (58.8)	84 (34.0)	
>1500 g (%)	55 (24.2)	56 (22.4)	138 (55.9)	
Small for GA (%)	47 (20.7)	18 (7.2)	22 (8.9)	0.001
**Mothers' characteristics**				
Health insurance for low financial income	17 (7.5)	12 (4.8)	7 (2.8)	0.07
Upper socio-demographic level	80 (35.2)	77 (30.8)	87 (35.2)	0.49
**Pregnancy**				
Prenatal corticosteroid treatment (%)	167 (73.6)	171 (68.4)	177 (71.7)	0.45
Multiple pregnancies (%)	64 (28.2)	87 (34.8)	78 (31.6)	0.30
Cesarean section (%)	177 (78.0)	167 (66.8)	130 (52.6)	0.001
Premature rupture of membranes >24 h	22 (9.7)	31 (12.4)	38 (15.4)	0.17
Hypertension during pregnancy	34 (15.0)	35(14.0)	36 (14.6)	0.96
**Neonatal hospitalization**				
Surfactant therapy (%)	121 (53.3)	125 (50.0)	102 (41.3)	0.25
Maternofetal sepsis (%)	38 (16.7)	38 (15.2)	38 (15.4)	0.88
Nosocomial sepsis (%)	68 (30.0)	56 (22.4)	43 (17.4)	0.005
Bronchopulmonary dysplasia (%)	18 (7.9)	5 (2.0)	7 (2.8)	0.002
Hemodynamic failure (%)	26 (11.5)	14 (5.6)	12 (4.9)	0.01
Patent ductus arteriosus requiring treatment (%)	51 (22.5)	34 (13.6)	18 (7.3)	0.001
**Outcome at 2 years**	*N* = 201	*N* = 219	*N* = 211	
Optimal outcome (%)	163 (81.1)	185 (84.5)	182 (86.3)	0.35
Impaired functional outcome (%)	38 (18.9)	34 (15.5)	29 (13.7)	

### Risk factors for impaired functional outcome

The results of the univariate analysis between neurological outcome and the characteristics of preterm infants are presented in Table 2. Impaired functional outcome was significantly associated with characteristics of neonatal hospitalization and decreased as GA and birthweight increased (p = 0.001).

**Table 2:** Association between neonatal characteristics of 631 infants and non-optimal neurological outcome at 2-year corrected age.

Variables	OR (95% CI)	p-value
**Infants' characteristics**		
Gestational age (GA)		
24–26 weeks	3.8 (1.9–7.8)	0.001
27–28 weeks	2.4 (1.3–4.1)	
29–30 weeks	1.2 (0.9–2.7)	
31–32 weeks	1.0	
Birthweight		
<1001 g	2,8 (1.6–5.0)	0.001
1001–1500 g	1.6 (0.9–2.8)	
>1500 g	1.0	
Small for GA	3.2 (1.9–5.3)	0.001
Male	1.4 (0.9–2.2)	0.14
**Mothers' characteristics**		
Health insurance for low financial income	2.8 (1.4–5.9)	0.005
Upper socio-demographic level	0.8 (0.5–1.3)	0.47
**Characteristics of pregnancy**		
Prenatal corticosteroid treatment	0.9 (0.6–1.5)	0.79
Multiple pregnancies	0.8 (0.5–1.3)	0.40
Cesarean section	1.4 (0.9–2.2)	0.19
Premature rupture of membranes >24 h	0.7 (0.4–1.3)	0.26
Hypertension during pregnancy	1.1 (0.6–2.1)	0.71
**Characteristics of neonatal hospitalization**		
Surfactant therapy	1.94 (1.2–3.0)	0.003
Maternofetal sepsis	1.99 (1.2–3.3)	0.007
Nosocomial sepsis	1.81 (1.1–2.9)	0.010
Bronchopulmonary dysplasia	2.96 (1.3–6.6)	0.005
Hemodynamic failure	2.72 (1.4–5.2)	0.002
Patent ductus arteriosus requiring treatment	1.82 (1.1–3.1)	0.025
**Cerebral radiologic assessment**		
No lesion	1.0	0.001
Intraventricular hemorrhage grade 1–2	1.14 (0.4–3.0)	
Intraventricular hemorrhage grade 3–4	9.12 (2.0–41.7)	
White matter lesions	8.31 (3.9–17.7)	
Radiologic assessment not available	1.67 (0.8–3.4)	

### Max-SBL and neurodevelopmental outcome

Impaired functional outcome was 19%, 15% and 14% in the first, second and third tertiles, respectively (p = 0.35). Adjustments for infant, mother, pregnancy and neonatal hospitalization characteristics did not reveal any association between max-SBL tertiles and neurodevelopmental outcome (Table 3). Additional adjustment for radiological brain lesions provided similar results (aOR for the second tertile: 1.0, 95% CI: 0.6–1.9, p = 0.95; aOR for the third tertile: 1.0, 95% CI:0.5–2.0, p = 0.92). Moreover, Max-SBL was not correlated with ASQ (n = 537, R^2^ adjusted for GA = 0.004, p = 0.64).

**Table 3:** Association between maximal serum non-conjugated bilirubin level and non-optimal neurological outcome at 2-year corrected age (n = 631).

Tertiles of maximal serum non-conjugated bilirubin levels and adjustment	OR (95% CI)	p-value
No adjustment		
First tertile	1	
Second tertile	0.8 (0.5–1.3)	0.36
Third tertile	0.7 (0.4–1.2)	0.16
Adjustment for infants' characteristics		
First tertile	1.0	
Second tertile	1.0 (0.6–1.8)	0.95
Third tertile	1.1 (0.6–2.0)	0.80
Adjustment for infant, mother and pregnancy characteristics		
First tertile	1.0	
Second tertile	1.0 (0.6–1.7)	0.97
Third tertile	1.1 (0.6–1.9)	0.86
Adjustment for infant, mother, pregnancy and neonatal hospitalization characteristics		
First tertile	1.0	
Second tertile	1.0 (0.6–1.8)	0.96
Third tertile	1.1 (0.6–2.0)	0.82

Pregnancy characteristics included prenatal corticosteroid treatment, multiple pregnancies, hypertension during pregnancy, premature rupture of membranes >24 h and cesarean section. Mother characteristics included health insurance for low financial income and upper socio-demographic level. Infant characteristics included GA, birthweight, small for GA and gender. Neonatal hospitalization characteristics included surfactant therapy, maternofetal sepsis, nosocomial sepsis, bronchopulmonary dysplasia, hemodynamic failure and patent ductus arteriosus requiring treatment.

In the subpopulation of 151 infants with birthweight <1001 g, neurological outcome tended to be different among the three tertiles (p = 0.05). Those with the most elevated max-SBLs (third tertile) had a greater tendency for an impaired functional outcome at 2-year corrected age (p = 0.05) (Table 4). Moreover, after adjustment for infant, mother, pregnancy, neonatal hospitalization characteristics and radiological brain lesions, the infants in the third tertile with a birthweight <1001 g had a significantly more elevated risk for impaired functional outcome than those in the second tertile with a birthweight <1001 g (aOR = 6.8, 95% CI:1.2–36.7, p = 0.03). Max-SBL was not correlated with ASQ (n = 128, R^2^ adjusted for GA = 0.006, p = 0.55). In contrast, we did not observe any difference between tertiles among infants with birthweight over 1000 g and Max-SBL. Moreover, Max-SBL was not significantly correlated with ASQ in infants with birthweight over 1500 g (n = 185, R^2^ adjusted for GA = 0.005, p = 0.56) and tended to be correlated in infants with birthweight between 1001 and 1500 g (n = 221, R^2^ adjusted for GA = 0.03, p = 0.09).

**Table 4:** Association between maximal non conjugated bilirubin level and non-optimal neurological outcome in 631 infants at 2-year corrected age (subpopulations studies).

POPULATIONS	N	Crude OR (95% CI)	p-value	aOR (95% CI)	p-value
**Birthweight <1001 g**	**151**				
First tertile	89	1		1	
Second tertile	41	0.4 (0.1–1.1)	0.09	0.4 (0.1–1.5)	0.17
Third tertile	21	2.2 (0.8–5.8)	0.12	3.1 (0.8–11.4)	0.09
**Birthweight from 1001 to 1500 g**	**268**				
First tertile	66	1		1	
Second tertile	127	1.2 (0.5–2.7)	0.70	1.9 (0.7–5.0)	0.18
Third tertile	75	0.9 (0.3–2.4	0.76	1.6 (0.5–4.7)	0.42
**Birthweight >1500 g**	**212**				
First tertile	46	1		1	
Second tertile	51	1.3 (0.4–4.4)	0.67	1.5 (0.4–5.8)	0.53
Third tertile	115	0.8 (0.3–2.4)	0.66	0.7 (0.2–2.7)	0.64

Adjustment was performed for pregnancy, infant and neonatal hospitalization characteristics. Pregnancy characteristics included prenatal corticosteroid treatment, multiple pregnancies, hypertension during pregnancy, premature rupture of membranes >24 h, and cesarean section. Mother characteristics included health insurance for low financial income and upper socio-demographic level. Infant characteristics included GA, birthweight, small for GA, gender. Neonatal hospitalization characteristics included surfactant therapy, maternofetal sepsis, nosocomial sepsis, bronchopulmonary dysplasia, hemodynamic failure and patent ductus arteriosus requiring treatment.

**Figure 2:**
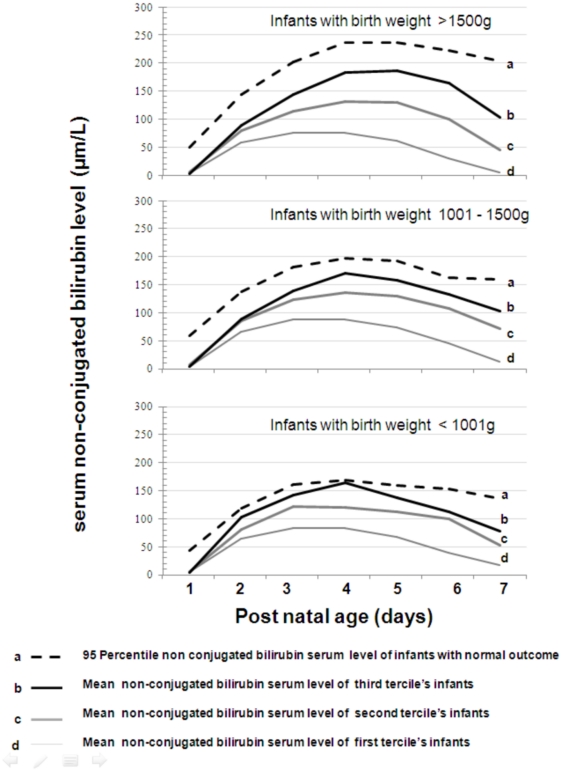
Changes in mean serum unconjugated bilirubin levels (SBL) during the first week of life in relation to birthweight. This figure shows three graphs: (1) the upper graph concerns infants with birthweight under 1500 g; (2) the intermediary graph concerns infants with birthweight between 1001 and 1500 g, and (3) the lower graph concerns the smallest infants (under 1001 g). Each graph is composed by four curves: three curves representing changes in mean SBL in infants of the first tertile (line a), second tertile (line b) and third tertile (line c). The fourth curve (dotted line or line d) represents the 95^th^ percentile of SBL in infants with an optimal neurological outcome at 2-year corrected age.

Mean SBLs for the first week of life were compared to the 95^th^ percentile curves of SBLs of infants with normal outcome at 2 years (Figure 2). Infants in the third tertile with birthweight under 1001 g presented a SBL curve that evolved relatively similarly to the 95^th^ percentile curve of SBLs of infants with a normal neurological outcome. The recommended max-SBLs (maximal levels of 95^th^ percentile curves of SBLs of infants with an optimal outcome) reached 170 µmol/L (9.9 mg/dL) when birthweight was under 1001 g, and 196 µmol/L (11.5 mg/dL) when birthweight varied between 1001 and 1500 g.

## Discussion

Our study of a large population-based cohort showed that premature infants born before 33 weeks of gestation, and for whom phototherapy was started in accordance with Maisels' recommendations, presented similar neurodevelopmental outcome at 2-year corrected age, irrespective of the max-SBL recorded during their first week of life. This study presents some limitations. One of them is that it concerned a group of mixed and poorly comparable premature infants born at 24 and 32 weeks of gestation. For this reason, we also performed a subgroup analysis in infants with birthweight under 1001 g. The infants at higher risk of impaired neurodevelopmental outcome presented lower max-SBL, because phototherapy was started at lower SBLs, according to Maisel's recommendations. In this subpopulation, we found that among infants with birthweight under 1001 g, those with elevated max-SBLs had a higher risk of developing impaired functional outcome, than those with low max-SBLs. However, only an interventional controlled trial could confirm this observation.

On the other hand, the strength of this retrospective report is that it was performed on a large sample and the follow-up rates were high.

Given the lack of validation for phototherapy guidelines in VLBW infants, our study raised critical questions that could affect daily clinical practice [13]. Until now, only one study analyzed the effect of Maisels' recommendations on neurological outcome [8]. In this study, among a population of infants under 1500 g or born before 32 weeks of gestation, the authors found a significant difference in 2-year neurodevelopmental outcome that depended on the infants' maximal total bilirubin levels. Nevertheless, the lack of transfontanellar ultrasound assessment could bias the results [8], [10]. In our cohort, the presence of brain lesions did not differ among the three groups, and no relationship between max-SBL and neurodevelopmental outcome was detected. However, the characteristics of the three tertiles differed widely, with younger and smaller infants in the first tertile. Although no difference was found among the groups in relation to outcome, we cannot exclude the possibility that we failed to adjust for all the risk factors associated with neonatal characteristics. The work reported by Oh *et al.* revealed an association between hyperbilirubinemia and poor outcome in unstable infants [12]. Nevertheless, only one sample (at five days of age) was collected, and the phototherapy procedure was different for each infant, taking into account that the cohort was built from a precedent study assessing two different phototherapy strategies [21].

Among infants weighing less than 1000 g, the difference in outcome suggested that a revision of Maisels' recommendations could be proposed, consisting in a decrease of the SBLs threshold. Because of physiological particulars and many comorbidity factors [3]–[4], it is difficult to define guidelines for starting phototherapy in VLBW infants. One validated method consists of analyzing data in infants with optimal outcome and then defining norms [22]. To determine the most adapted SBL for starting phototherapy in infants under 1001 g, we compared the 95^th^ percentile of SBLs in infants with optimal neurological outcome in relation to birthweight. The maximal level of these 95^th^ percentile curves differed by 26 µmol/L between infants under 1001 g (170 µmol/l or 10 mg/dL) and those with a birthweight between 1001 and 1500 g (196 µmol/l or 11.5 mg/dL). Consequently, a reduction of 26 µmol/L (1.5 mg/dL) from Maisels' threshold can be suggested for infants under 1001 g, i.e., 114 µmol/L (6.7 mg/dL) rather than 140 µmol/L (8.2 mg/dL), when no hemolytic risk factors are present. A recent report by Moll *et al.* on two ELBW infants with kernicterus supports our recommendation, despite supposed moderate hyperbilirubinemia [13].

Intensification of phototherapy in younger, premature infants is subject to debate. In 1992, O'Shea *et al.* studied the relationship between maximal total bilirubin and neurodevelopmental outcome in infants with birthweight under 1500 g with a total bilirubin threshold for phototherapy of 86 µmol/L (5 mg/dL) [9]. This management of hyperbilirubinemia seemed safe and efficient. No association between maximal total bilirubin and neurodevelopmental outcome at 1-year corrected age was found. Morris *et al.* compared aggressive and conservative phototherapy in 1,974 ELBW infants [21], in whom aggressive phototherapy was started for total bilirubin levels higher than 86 µmol/L (5 mg/dL), and conservative phototherapy was started for total bilirubin levels higher than 137 µmol/L (8 mg/dL) (subgroup 501–750 g) or 171 µmol/L (10 mg/dL) (subgroup 751–1000 g). The rate of death or neurodevelopmental impairment was not significantly reduced by aggressive phototherapy. Conversely, aggressive phototherapy was associated with increased mortality in infants with birthweight between 501 and 750 g. The reduction of antioxidant protection in aggressive phototherapy could explain this result, as bilirubin presents antioxidant effects [2] and phototherapy may cause oxidative injury by photodegradation of antioxidant molecules [23]–[24].

Thus, the difference in neurodevelopmental outcome among infants weighing less than 1001 g in our study could be due to the differences in max-SBL and to the duration of phototherapy. The infants with higher max-SBL and, consequently, with a more important exposure to phototherapy, could present a deficit in antioxidant components at the origin of a poorer neurodevelopmental outcome. The difference in impaired functional outcome in infants weighing under 1001 g was very significant when we compared them with those with high max-SBLs (third tertile) and those with moderate max-SBLs (second tertile). Lower SBLs in the first tertile could have limited the protective antioxidant effect of bilirubin and reduced the difference in the third group.

In conclusion, we did not observe any difference in impaired functional outcome between the three tertiles of max-SBL, except for infants under 1001 g. Although there are inherent limits to this type of observational studies, our data tend to validate Maisels' recommendations in the global population of neonates born before 33 weeks of gestation, except for infants with birthweight lower than 1001 g, suggesting the necessity for a revision of the phototherapy threshold for infants with VLBW.
